# Auditory cortical delta-entrainment interacts with oscillatory power in multiple fronto-parietal networks

**DOI:** 10.1016/j.neuroimage.2016.11.062

**Published:** 2017-02-15

**Authors:** Anne Keitel, Robin A.A. Ince, Joachim Gross, Christoph Kayser

**Affiliations:** Institute of Neuroscience and Psychology, University of Glasgow, 58 Hillhead Street, Glasgow G12 8QB, UK

**Keywords:** Auditory entrainment, Speech processing, MEG, Delta band, Prosodic parsing

## Abstract

The timing of slow auditory cortical activity aligns to the rhythmic fluctuations in speech. This entrainment is considered to be a marker of the prosodic and syllabic encoding of speech, and has been shown to correlate with intelligibility. Yet, whether and how auditory cortical entrainment is influenced by the activity in other speech–relevant areas remains unknown. Using source-localized MEG data, we quantified the dependency of auditory entrainment on the state of oscillatory activity in fronto-parietal regions. We found that delta band entrainment interacted with the oscillatory activity in three distinct networks. First, entrainment in the left anterior superior temporal gyrus (STG) was modulated by beta power in orbitofrontal areas, possibly reflecting predictive top-down modulations of auditory encoding. Second, entrainment in the left Heschl's Gyrus and anterior STG was dependent on alpha power in central areas, in line with the importance of motor structures for phonological analysis. And third, entrainment in the right posterior STG modulated theta power in parietal areas, consistent with the engagement of semantic memory. These results illustrate the topographical network interactions of auditory delta entrainment and reveal distinct cross-frequency mechanisms by which entrainment can interact with different cognitive processes underlying speech perception.

## Highlights

• We study auditory cortical speech entrainment from a network perspective.
• Found three distinct networks interacting with delta-entrainment in auditory cortex.
• Entrainment is modulated by frontal beta power, possibly indexing predictions.
• Central alpha power interacts with entrainment, suggesting motor involvement.
• Parietal theta is modulated by entrainment, suggesting working memory compensation.

## Graphical abstract

**Figure:**
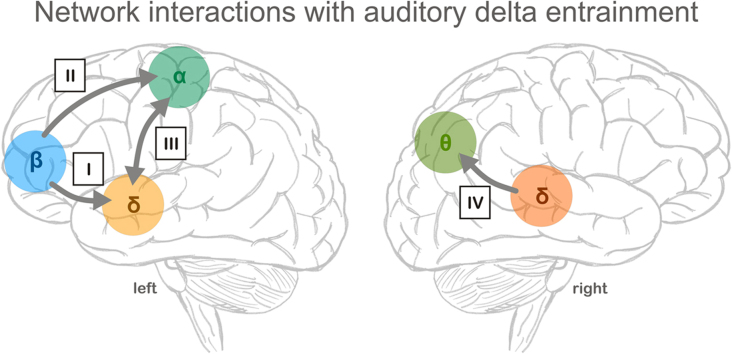

## 1 Introduction

While listening to speech, rhythmic auditory cortical activity aligns to the quasi-rhythmic regularities arising from stress, syllabic rate, or phonemes. This entrainment of brain activity to speech is particularly prominent in the delta (below 4 Hz) and theta (4–8 Hz) frequency bands (Ahissar et al., 2001, Aiken and Picton, 2008, Di Liberto et al., 2015, Giraud and Poeppel, 2012, Gross et al., 2013, Kayser et al., 2015, Luo and Poeppel, 2007, Ng et al., 2012) and is particularly strong in the auditory cortex (Aiken and Picton, 2008, Gross et al., 2013). Given that the degree of entrainment is predictive of speech intelligibility and comprehension rates (Ahissar et al., 2001, Ding et al., 2014, Ghitza et al., 2012, Ghitza and Greenberg, 2009, Luo and Poeppel, 2007, Peelle and Davis, 2012, Peelle et al., 2013), the alignment of auditory cortical activity to the speech envelope has been proposed to subserve a number of important functions, such as the parsing or encoding of acoustic and phonological features (Cogan and Poeppel, 2011, Ghitza, 2013, Ghitza et al., 2012, Giraud and Poeppel, 2012, Howard and Poeppel, 2010, Peelle and Davis, 2012), or the selection of sensory streams (Schroeder and Lakatos, 2009). While auditory entrainment is frequently used as a marker for speech encoding, the network-level interactions that shape the underlying neural processes remain unknown.

Language processing depends on a large network of interconnected brain areas (Bornkessel-Schlesewsky et al., 2015, Friederici and Gierhan, 2013, Hickok and Poeppel, 2007, Poeppel, 2014, Rauschecker and Scott, 2009), with ventral and dorsal pathways linking auditory cortex with fronto-parietal regions implicated in extracting acoustic, lexical or categorical information (Alho et al., 2014, Bornkessel-Schlesewsky et al., 2015, Friederici and Gierhan, 2013, Hickok and Poeppel, 2007, Schomers et al., 2015, Smalle et al., 2015, Wilson et al., 2004). Interestingly, many studies have shown that auditory perception depends on the state of rhythmic activity, such as theta, alpha or beta activity, in auditory or frontal regions (Henry et al., 2014, Henry and Obleser, 2012, Neuling et al., 2012, Ng et al., 2012, Strauss et al., 2015). Furthermore, oscillatory power in different frequency bands and fronto-parietal brain regions has been quantified in numerous studies investigating cognitive control or attention (de Lange et al., 2008, Hipp et al., 2011, Schroeder and Lakatos, 2009, Stoll et al., 2016, van de Vijver et al., 2011). In the context of speech perception, the state of alpha power has been found to co-vary with experimental manipulations such as attention (Kelly et al., 2006, Klimesch et al., 1998), listening effort (Obleser et al., 2012), and speech intelligibility (Obleser and Weisz, 2012). In addition, changes in theta power have been linked to lexical retrieval processes and semantic working memory (Bastiaansen and Hagoort, 2006, Bastiaansen et al., 2005), while changes in beta and gamma power have been associated with a predictive coding framework, representing top-down predictions and bottom-up prediction errors during language processing (Arnal and Giraud, 2012, Arnal et al., 2011, Lewis and Bastiaansen, 2015). However, the functional interpretation of the processes indexed by the oscillatory activity extracted from fronto-parietal regions usually rests on the implicit assumption that changes in this oscillatory activity correlate with, or even causally relate to, changes in the auditory cortical speech representations provided by the entrainment of neural activity to the speech envelope (Arnal and Giraud, 2012, Kayser et al., 2015, Park et al., 2015). For example, changes in frontal alpha during enhanced listening effort have been suggested to relate to changes in the precision of auditory cortical entrainment (Kayser et al., 2015). However, no study to date has directly tested the hypothesis that changes in fronto-parietal oscillatory processes directly correlate with the fidelity of dynamic speech representations in temporally entrained auditory cortical activity.

To address the functional dependence between auditory speech entrainment and the state of oscillatory activity in fronto-parietal regions, we used source-localised MEG data obtained while participants listened to a continuous story (Gross et al., 2013). We chose an unbiased approach, in that we did not limit our analyses to specific and pre-defined anatomical regions of interest within frontal or parietal lobes. The reason for this was that previous studies have implied a vast number of regions in language processes, or provided only very coarse localizations of these. Likewise, we included a wide range of frequency bands when assessing the state of oscillatory activity in fronto-parietal regions (from delta to gamma), as previous studies have implied a wide range of rhythmic processes in speech perception. As a result, we systematically quantified the relation between auditory cortical speech entrainment and states of oscillatory power in fronto-parietal regions across a wide range of frequency bands.

## 2 Materials and methods

### 2.1 Participants and data acquisition

MEG data were acquired from 23 healthy, right-handed participants (12 female, mean age 26.9±7.9 years [*M*±*SD*]) as part of a previous study (Gross et al., 2013). All participants provided written informed consent prior to testing. The experiment was approved by a local ethics committee (University of Glasgow, Faculty of Information and Mathematical Sciences), and conducted in compliance with the Declaration of Helsinki.

MEG-recordings were obtained with a 248-magnetometers whole-head MEG system (MAGNES 3600 WH, 4-D Neuroimaging; sampling rate: 1017 Hz). The participants’ head positions were measured at the beginning and end of each run via 5 coils placed on the forehead and behind the ears. Head position was co-digitised with head-shape (FASTRAK®, Polhemus Inc., VT, USA). Participants sat upright and fixated a cross projected centrally on screen with a DLP projector while listening to two auditory presentations in a pseudo-randomised order. Sounds were presented binaurally via plastic earpieces and 5-m long plastic tubes connected to a sound pressure transducer. Stimulus presentation was controlled with Psychtoolbox (Brainard, 1997) for MATLAB (The MathWorks, Inc.).

### 2.2 Auditory stimuli

The ‘forward’ story condition consisted of an approximately 7-min long narration (‘Pie-man’ told by Jim O’Grady, recorded at ‘The Moth’ storytelling event in New York, 2012). This narration comprises approximately 950 words, with syllables spoken at an average rate of 6.8 Hz. This is in line with studies that establish the syllabic rate typically in the theta-range (4–8 Hz) (Cotton, 1936, Hyafil et al., 2015, Poeppel, 2003). In natural speech, the rate of prominent, or stressed, syllables is approximately one third of all syllables (e.g., Kochanski et al., 2005), essentially placing it in the delta-range (1–4 Hz) (Goswami and Leong, 2013, Greenberg et al., 2003). Consequently, entrainment in the delta band has been proposed to reflect prosodic fluctuations in speech (Ghitza, 2013, Ghitza et al., 2012). The ‘backward’ condition consisted of the same story played backwards and served as an unintelligible control condition.

### 2.3 Extraction of speech envelope

From the waveform of the acoustic stimulus we computed the wideband speech envelope by band-pass filtering (3rd order Butterworth filter, forward and reverse) into eight bands in the range of 100–10,000 Hz, equidistant on the cochlear frequency map (Smith et al., 2002). Individual band-limited envelopes were obtained using the magnitude of the Hilbert transform and were subsequently averaged to obtain the wide band speech envelope (Chandrasekaran et al., 2009, Drullman, 1995, Gross et al., 2013, Kayser et al., 2015, Smith et al., 2002). The envelope was resampled to 150 Hz for subsequent analysis.

### 2.4 MEG data processing

Data were analysed using MATLAB (The MathWorks, Inc.), including external toolboxes, such as FieldTrip (Oostenveld et al., 2011), and custom-written routines. The MEG signal was detrended and resampled to 150 Hz. Data were band-limited to seven frequency bands (*delta* 1–4 Hz, *theta* 4–8 Hz, *alpha* 8–12 Hz, *low beta* 12–18 Hz, *beta* 18–24 Hz, *high beta* 24–36 Hz, *gamma* 30–48 Hz), using FIR filters (forward and reverse, with 60 dB stop-band attenuation, 1-Hz transition bandwidth, and 0.01 dB pass-band ripple).

### 2.5 MEG source localisation

Individual, T1-weighted structural magnetic resonance images (MRIs) were manually co-registered to the MEG coordinate system by using participants’ digitised head shapes. MRIs were further realigned with individual head shapes through an iterative closest point (ICP) algorithm (Besl and Mckay, 1992). MRIs were then segmented to obtain a representation of the brain, including grey and white matter, and cerebrospinal fluid. A single-shell model was used to construct a volume conduction model (Nolte, 2003). Individual anatomical MRIs were linearly transformed to a template (MNI) brain using Fieldtrip/SPM5. Sensor level data were transformed into source space using the linear constraint minimum variance (LCMV) beamformer (Van Veen et al., 1997) on a 4-mm regular grid covering the entire brain (7% regularisation). The optimal orientation for each dipole was computed using the SVD approach. We used the AAL atlas (Automated Anatomical Labelling atlas) to parcellate the template brain into 116 anatomical areas (Tzourio-Mazoyer et al., 2002). As the superior temporal gyrus (STG) comprises a very large and functionally differentiated area in the AAL atlas, we divided it further into an anterior and posterior section (e.g., Friederici, 2002; Hickok and Poeppel, 2007). Specifically, the median of voxel positions along the horizontal plane (i.e., y-coordinates in MNI space) was used as threshold for the division between anterior and posterior parts to obtain equally-sized anterior and posterior STG partitions.

### 2.6 Region-specific analyses

**Fig. 1:**
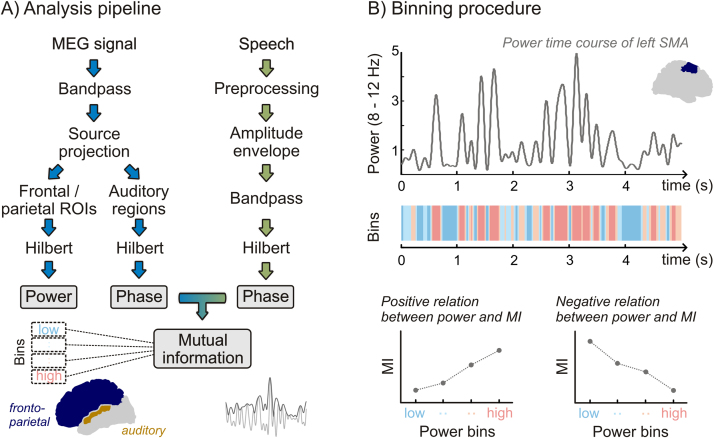
Quantifying the dependency between auditory speech entrainment and the activity state in fronto-parietal regions. A) General analysis strategy. After preprocessing the acoustic waveform (including filtering in narrow bands, equidistant on the cochlear frequency map), the broadband speech amplitude envelope was derived. Speech and MEG signals were bandpass filtered into frequency bands and the MEG signal was projected into source space. The Hilbert transform was used to derive the instantaneous phase and power. Anatomical regions of interest (ROIs) were extracted using the AAL atlas. For each fronto-parietal ROI (coloured in blue), the oscillatory power was divided into four bins. For grid points within auditory regions (coloured in dark orange), the mutual information between the phase of the local oscillatory activity and the phase of the speech envelope was computed separately for each power state (bin) derived from each fronto-parietal ROI (ROIs closer than 3.5 cm to the respective auditory region were excluded). B) Binning procedure. Upper panel: Example of the time course of alpha power in the left supplementary motor area (inlet shows midsagittal view of left hemisphere). Middle panel: The same time course segmented into bins from low (blue) to high (red) power. MI in auditory areas was calculated separately for time points falling into each power bin, resulting in four MI values for each fronto-parietal ROI, power frequency band (delta to gamma), auditory region and auditory frequency band (delta/theta). MI-values were then regressed on a linear predictor (values 1 to 4, representing low to high power states). Lower panel: Illustration of hypothetical positive and negative relations between MI and power state (low to high).

We quantified the entrainment of rhythmic activity to speech within three auditory regions in each hemisphere (Heschl's Gyrus, anterior/posterior superior temporal gyrus [aSTG/pSTG]). For each auditory region, the bandpass-filtered MEG source-space data were Hilbert-transformed to derive the instantaneous phase for each time and grid point (for an analysis overview, see Fig. 1A).

We quantified the state of oscillatory activity in 46 frontal, central, and parietal ROIs. These included all ROIs of the AAL atlas in frontal and parietal lobes, motor regions, rolandic operculum, cingulate and angular gyri, insula, cuneus and precuneus. For these fronto-parietal ROIs, bandpass-filtered signals were Hilbert-transformed to derive the instantaneous power for each time and grid point. The power at each grid point was normalised for each frequency band by its time average, and the normalised power was then averaged across grid points to obtain a power time series for each ROI.

### 2.7 Speech entrainment quantified by mutual information

To quantify the statistical dependency between the speech envelope and the MEG source data, we used mutual information (MI) (Gross et al., 2013, Kayser et al., 2015). MI measures how much knowing one signal reduces the uncertainty about another signal and is expressed on a common principled scale in units of bits. MI values between two time series were calculated, using a robust bin-less approach based on the concept of statistical copulas (for details, see Ince et al., 2016, 2015; Kayser et al., 2015). When using phase as a variable, the phase was expressed as a unit magnitude complex number. The real and imaginary parts were standardised separately and combined as a two-dimensional variable for the MI calculation.

For an initial analysis, we computed within-band MI between the instantaneous phase of the source signal at each grid point and the instantaneous phase of the speech amplitude envelope, separately for each frequency band from delta to gamma. The MI was computed using a frequency-specific time-lag between the speech envelope and the MEG data. This was estimated by computing phase coherence for each participant between the acoustic amplitude envelope and the MEG time series within frequency bands for each grid point in auditory areas, using different lags from 1 to 30 sampling points (i.e. up to a lag of 200 ms), in steps of 1 sampling point. Phase coherence was averaged across grid points within auditory regions. The lag with maximum phase coherence was then chosen and averaged across participants (e.g., 140 ms for delta, and 147 ms for theta). MI was computed including all grid points for the whole-brain analysis. For subsequent analyses, MI was analysed for each grid point in auditory areas and then averaged within each auditory region.

### 2.8 Dependence of auditory cortical speech MI on the power in individual ROIs

To quantify whether the speech MI in auditory regions was related to the state of oscillatory power in the fronto-parietal ROIs, we binned the time series of the power in each of the 46 ROIs into four equipopulated bins representing different levels of amplitude (see Fig. 1B). For each auditory region, speech MI was then calculated separately for time points corresponding to each power bin (‘state’). Linear dependencies of MI on power states were analysed using within-subject regression analysis, with positive dependencies reflecting higher MI for higher power. Regression was performed for MI values in each of the six auditory regions in two frequency bands (delta, theta), in dependency on power in 46 fronto-parietal ROIs in seven power bands (delta to gamma). Group-level analysis was then performed on the *t*-statistics of the regression of betas using a cluster-based permutation approach (Maris and Oostenveld, 2007), correcting for multiple comparisons across frequency bands and fronto-parietal ROIs. Cluster statistics were derived separately for each auditory region (detailed parameters: 2000 iterations, including only bins with a two-tailed alpha of *p*<.025 in the cluster analysis, performing a two-tailed *t*-test at *p*<.05 on the clustered data; using the 3D-distance between ROI centres as neighbourhood information). Effect sizes for cluster-based *t*-statistics are reported as the summed *t*-value across all bins (fronto-parietal ROIs, power bands, and phase bands) within a cluster (*T*_sum_). For the *backward* condition, cluster statistics yielded no comparable effects for which *T*_sum_- or *p*-values could be reported. Instead, we report *t*-statistics with FDR-corrected *p*-values for these ROIs and frequency bands that were significant in the forward condition. To avoid spurious effects from signal blurring in source space or overlap between frequency bands we excluded fronto-parietal ROIs that were adjacent (<3.5 cm centre to centre) to the analysed auditory region, and we excluded within-frequency comparisons from the cluster statistics (i.e., comparison of entrainment and power state within the same band).

### 2.9 Directionality of MI-dependence on power

To determine whether the relation between auditory speech entrainment and fronto-parietal oscillatory power is temporally directional, we repeated the above regression analysis by systematically testing different temporal lags between the time points used to compute speech MI and oscillatory power – similar to other directional approaches such as Granger causality (Granger, 1969) or transfer entropy (Schreiber, 2000). For this analysis, time points for each ROI belonging to a cluster were again binned according to the four power states, but instead of computing speech MI at corresponding time points, it was computed at different time points before and after the sampling points of oscillatory states (shifted between -200 ms and 200 ms in steps of 1 sampling point, i.e., ~6.67 ms). Speech MI was then averaged within each ROI cluster. The regression analysis between speech MI and power states was repeated for each lag (where zero lag represents the original result). We then tested whether the time points of peak effects in the group-level regression statistics were significantly different from zero, using a jackknife test that reduces the bias in the estimate of the population value (Tukey's jackknife; see Sokal and Rohlf, 1995, pp. 820–821, for used formulas). For this procedure, the leave-one-out jackknife distribution is first z-transformed, the values then transformed into “pseudovalues”, and the statistics are based on these Jackknife estimates. Note that the jackknifed estimates of the statistics can differ from leave-one-out means, as they represent bias-free estimates. A positive peak effect indicates that speech-brain entrainment is related to the oscillatory power in preceding time bins.

### 2.10 Entrainment between speech and ROI clusters

For those fronto-parietal ROIs that exhibited a significant relation to auditory entrainment in our main analysis, we also computed the degree of speech entrainment of their oscillatory activity. For this, the MI was calculated between the power time course of each ROI and the delta phase of the speech envelope and then averaged within each ROI cluster. To estimate the significance of MI values, we obtained a surrogate distribution of MI values under the null-hypothesis of no systematic alignment of MEG data to speech. Practically, we implemented this by randomly shifting the speech time series (circular shift with lag drawn from a uniform distribution on [1 N] where N is the number of samples in the time series) and calculating a distribution of 1000 surrogate MI values for each auditory region or fronto-parietal ROI cluster. To account for multiple comparisons across multiple ROIs, maximum statistics were used (Holmes et al., 1996).

### 2.11 Phase-amplitude coupling between ROI clusters and auditory regions

Phase-amplitude coupling was calculated as the magnitude of the time-averaged complex product of fronto-parietal power and auditory phase (for each grid point in auditory regions) (Canolty et al., 2006). Coupling values were then averaged within each cluster. For statistical analysis, we derived a surrogate distribution of 1000 phase-amplitude values, using randomly shifted time series (circular shift without limitations as to the lag), and compared the actual values with the percentiles of the group-level surrogate distribution.

### 2.12 Independence of effects for modulation of MI in aSTG

As the speech MI within the left anterior STG was related to alpha power in central ROIs and beta power in frontal ROIs, we asked whether these two effects were related to each other. To test for an interaction between alpha and beta power on entrainment, we entered both alpha and beta power states into a 4×4 ANOVA (four alpha power bins×four beta power bins) with delta MI in aSTG as dependent variable. Significant main effects would essentially replicate results of the regression analysis, whereas a significant interaction would suggest a dependent modulation of aSTG-entrainment by central alpha and frontal beta power. As a next step, we calculated within-subject Pearson correlations and cross-correlations between average beta and alpha power time series, to determine whether both signals were directly related. The resulting mean correlation coefficients were again compared to a surrogate distribution. The time points of the peaks in the cross-correlation (with lags between ±200 ms, in steps of 1 sampling point, i.e., ~6.67 ms), were compared to zero by means of a dependent-samples *t*-test.

## 3 Results

### 3.1 Entrainment of auditory cortical activity to the speech envelope

**Fig. 2:**
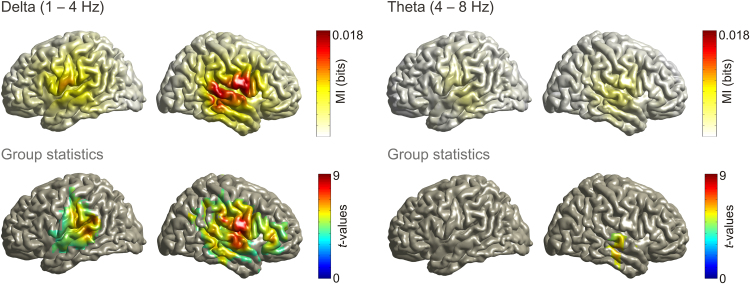
Entrainment in delta and theta frequency bands. Upper panel shows raw MI values averaged across participants. Lower panel shows significant grid points in comparison with backward-condition (*p*<.05 dependent samples *t*-test, FDR corrected).

We found significantly stronger speech MI for the forward condition, compared with the backward condition, in the *delta* (1–4 Hz) and *theta* (4–8 Hz) frequency bands within left and right auditory regions (group statistics, *p*<.05, false discovery rate [FDR] corrected, see Fig. 2). We did not observe significant differences between forward and backward conditions in higher frequency bands. Furthermore, MI values were higher for the delta compared to the theta band for all six auditory regions (all *p*_FDR_<.001, dependent-samples *t*-test; means across auditory regions: MI_delta_=0.008, MI_theta_=0.003). As our main goal was to quantify whether this entrainment is modulated by the state of activity within fronto-parietal ROIs, we limited the following analysis to the speech entrainment in delta and theta bands.

To quantify whether auditory entrainment was systematically related to changes in the power of oscillatory activity over fronto-parietal regions, we used group-level cluster-based regression to extract whether and for which auditory regions there was a significant linear dependency between speech MI and the state of oscillatory power in clusters of fronto-parietal ROIs over time within each participant (termed ‘ROI cluster’). This analysis was repeated separately for auditory delta and theta entrainment, and for seven frequency bands indexing the state of fronto-parietal activity. This analysis revealed four patterns of dependencies, which we describe in the following.

**Fig. 3:**
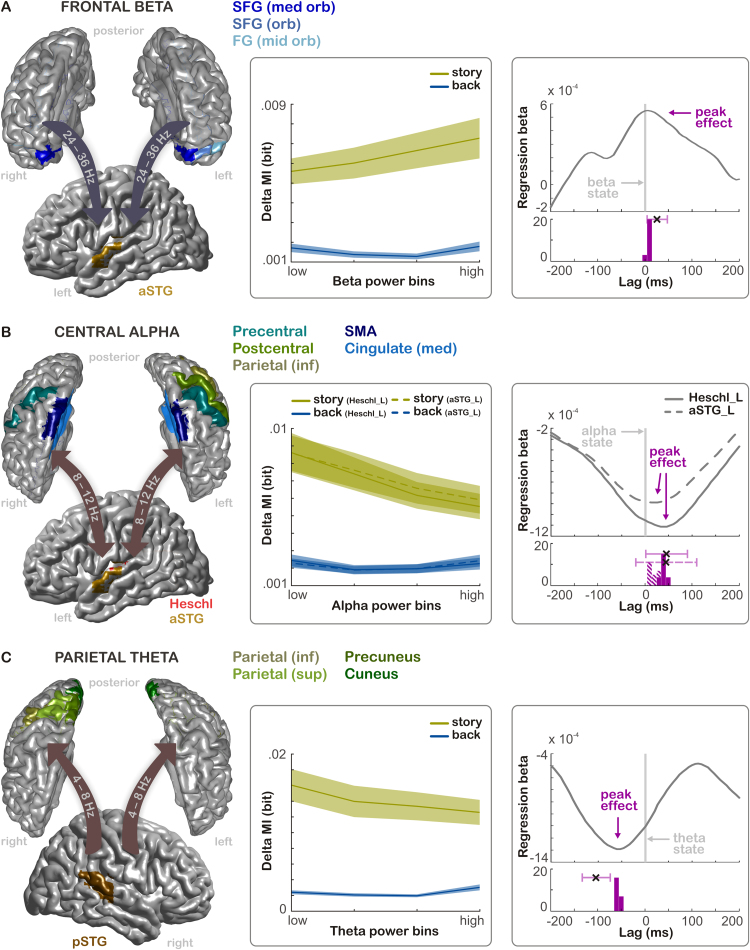
Dependence between auditory speech entrainment and activity states in fronto-parietal ROIs. (A)*(Left column)* Top-down modulation of delta MI in the left aSTG by beta power in orbito-frontal ROIs (bilateral medial orbital parts of the superior frontal gyrus, left superior orbito-frontal gyrus, left middle orbito-frontal gyrus). *(Middle column)* Delta MI as a function of binned beta power for the forward and backward speech conditions. Solid lines represent the average MI across participants, the shaded area the SEM across participants. The effect of beta power was significant for the story (*p*_cluster_=.04) but not the backward condition (*p*_FDR_=.39, no cluster found). *(Right column)* Dependence of the MI-modulation on the time-lag between power state and the time of MI calculation. The line in the upper panel shows the mean regression beta for this cluster (across participants) as a function of lag. The lower panel shows a histogram of the leave-one-out jackknife values and the associated mean (black cross) and 95% confidence interval (horizontal bar) derived from the jackknife statistics. The peak lag (at 7 ms) was significantly different from zero (*p*_FDR_<.05). (B)*(Left column)* Top-down modulation of delta MI in two auditory regions by alpha power in central/motor ROIs. MI in left Heschl's Gyrus was dependent on alpha power in bilateral SMA, bilateral precentral gyri, bilateral median cingulate gyri, and left postcentral gyrus. MI in left aSTG was dependent on power in the bilateral SMA, left precentral gyrus, bilateral median cingulate gyri, left postcentral gyrus, and left inferior parietal gyrus). *(Middle column)* The effect of alpha power was significant for the story (Heschl: *p*_cluster_<.001, aSTG: *p*_cluster_<.01) but not the backward condition (Heschl: *p*_FDR_=.39, aSTG: *p*_FDR_=.39, no cluster found). *(Right column)*. The peak lags (at 40 ms and 20 ms, respectively) were marginally significantly different from zero in Heschl's Gyrus (*p*_FDR_=.06), but not the aSTG (*p*_FDR_=.16). Dashed lines/bars illustrate effects of aSTG. (C)*(Left column)* Bottom-up modulation of theta power in parietal ROIs by delta MI in the right posterior STG. This cluster comprises the bilateral cuneus, right precuneus, right superior parietal gyrus, and part of the right inferior parietal lobule. *(Middle column)* The effect of theta power was significant for the story (*p*_cluster_=.01) but not the backward condition (*p*_FDR_=.24, no cluster found). *(Right column)* The peak lag (at -60 ms) was significantly different from zero (*p*_FDR_<.001). SFG, superior frontal gyrus; FG, frontal gyrus; SMA, supplementary motor area; aSTG, anterior superior temporal gyrus.

In the left anterior superior temporal gyrus (aSTG), delta MI was positively dependent on high beta power (24 – 36 Hz) in a cluster comprising bilateral superior frontal gyrus (medial orbital part), left superior frontal gyrus (orbital part), and left middle frontal gyrus (orbital part) (*frontal beta effect*, *p*_cluster_=.04, *T*_sum_=10.48, Fig. 3A). Fig. 3A, **middle column** illustrates the increase in delta MI with increasing beta power in this cluster. There was no significant influence of beta power on delta MI in the backward speech condition (t(22)=0.19, *p*_FDR_=.39).

In Heschl's Gyrus, as well as in the left anterior temporal gyrus (aSTG), delta MI was negatively dependent on alpha power (8–12 Hz). The two ROI clusters were highly overlapping. For Heschl's Gyrus, the cluster comprised bilateral supplementary motor areas, bilateral precentral gyri, bilateral median cingulate gyri, and left postcentral gyrus (*central alpha effect 1*, *p*_cluster_<.001, *T*_sum_=-25.02 (Fig. 3**B).** For the aSTG, the cluster comprised bilateral supplementary motor areas, left precentral gyrus, bilateral median cingulate gyri, left postcentral gyrus, and left inferior parietal gyrus (*central alpha effect 2*, *p*_cluster_=.002, *T*_sum_=-20.42. Delta MI in these auditory areas decreased with increasing alpha power in these ROIs. In the backward speech condition, there was no significant modulation of MI by alpha power (left Heschl's Gyrus: *t*(22)=-0.41, *p*_FDR_=.39; left aSTG: *t*(22)=0.44, *p*_FDR_=.39, Fig. 3**B, middle column**).

In the right posterior temporal gyrus (pSTG), delta MI was negatively dependent on theta power (4 – 8 Hz) in a cluster comprising bilateral cuneus, right precuneus, right superior parietal gyrus, and part of the right inferior frontal lobule (*parietal theta effect*, cluster statistics: *p*_cluster_=.01, *T*_sum_=-16.02 (Fig. 3**C).** With decreasing theta power in these parietal ROIs, delta MI in right pSTG increases. Again, there was no significant MI modulation in the backward speech condition, *t*(22)=1. 89, *p*_FDR_=.24 (Fig. 3**C, middle column**).

### 3.2 Directionality of speech-MI modulation

To probe the directionality of the relation between auditory entrainment and fronto-parietal power, we repeated the regression analyses by varying a relative temporal lag between the time points at which ROI power and speech MI were calculated. We used Jackknife resampling to test whether the peak of the group-level MI dependence on power was significantly different from the zero lag.

The modulation of delta entrainment in left aSTG by frontal beta power peaked 7 ms *after* the power state and this was significantly different from zero (95% CI [4,48] ms, *p*_FDR_<.05; *p*-values for this analysis are FDR-corrected across comparisons for each ROI cluster). The modulations of delta entrainment in left Heschl's Gyrus and aSTG by central alpha power peaked at 40 ms and 20 ms respectively, *after* the power state. In Heschl's Gyrus, this peak was marginally different from zero, whereas in aSTG it was not (Heschl: 95% CI [2,91] ms, *p*_FDR_=.06; aSTG: 95% CI [-20, 111] ms, *p*_FDR_=.16). Finally, the modulation of delta entrainment in right pSTG by parietal theta power peaked at 60 ms *before* power computation and was significantly different from zero (95% CI [-135, -75], *p*_FDR_<.001). These results suggest that the modulation of parietal theta power follows the speech entrainment in auditory regions, while modulations of central alpha and frontal beta power precede auditory speech entrainment and therefore likely reflect top-down influences on auditory encoding.

### 3.3 Brain – speech entrainment in fronto-parietal ROIs

**Fig. 4:**
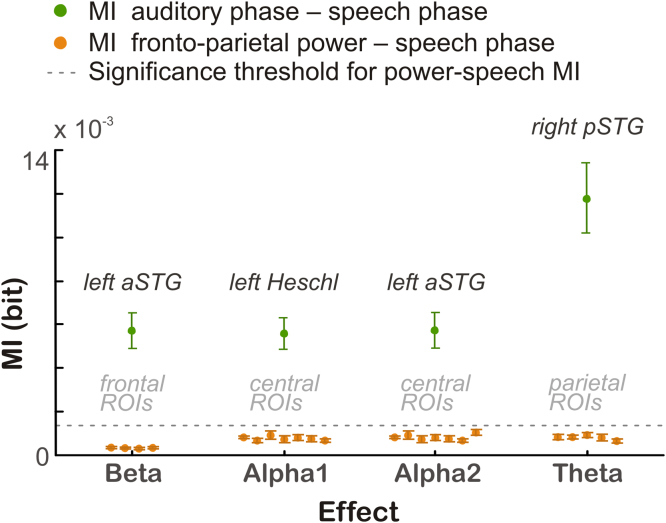
Brain-speech entrainment in fronto-parietal and auditory areas. MI between the *power* in fronto-parietal ROIs and the *delta phase* of the speech amplitude envelope (orange), and between the *delta phase* in auditory regions and the *delta phase* of the speech envelope (green). For fronto-parietal ROIs, MI values are represented as mean across participants for each individual ROI belonging to a cluster. Bars illustrate SEM across participants. Dotted line denotes significance level at 95% derived from surrogate data.

We ruled out that the dependence of speech MI on fronto-parietal activity trivially arises because fronto-parietal activity is by itself significantly entrained to the speech envelope. We calculated MI between the power in each of the fronto-parietal clusters and the delta phase of the speech amplitude envelope, which was the acoustic component driving the speech entrainment in the auditory regions. Speech MI in fronto-parietal power was not significant for any of the four clusters (*frontal beta*: Student's *t*=1.16, *p*_FDR_=.87, *central alpha 1* [Heschl's Gyrus]: Student's *t*=0.17, *p*_FDR_=.76, *central alpha 2* [aSTG]: Student's *t*=-0.68, *p*_corrected_=. 51, *parietal theta*: Student's *t* =-0. 93, *p*_FDR_=.51) and the MI values were considerably lower than those for the delta phase of the activity in auditory regions (Fig. 4).

### 3.4 Phase-amplitude coupling between ROI clusters and auditory regions

We also ruled out that the relation between delta entrainment in auditory regions and fronto-parietal power reflects a coupling of these signals during listening to speech that is independent of the acoustic input. Previous studies have described wide-spread coupling between the oscillatory phase at lower frequencies and the power at higher frequencies within and between brain regions, termed phase-amplitude coupling (e.g., Gross et al., 2013; Jensen and Colgin, 2007). Thus, we calculated the phase-amplitude coupling between auditory delta phase and the power in each of the four clusters. For no cluster did the observed phase-amplitude coupling values (*PAC*) reach statistical significance: *frontal beta* cluster (*PAC*=0.013; Student's *t*=-1.00, *p*_FDR_=.66); two central clusters (*central alpha 1*: *PAC*=0.011; Student's *t*=0.88, *p*_FDR_=.81; *central alpha 2*: *M*=0.012; Student's *t*=0.57, *p*_FDR_=.81); and *parietal theta* cluster (*PAC*=0.015; Student's *t*=-0.41, *p*_FDR_=.69).

### 3.5 Dependent influences of alpha and beta power on aSTG entrainment

As speech MI in the left anterior superior temporal gyrus was dependent on two distinct ROI clusters (negative dependence on central alpha and positive dependence on frontal beta-power), we asked whether these effects were statistically distinct. We entered both alpha and beta power as two factors into an analysis of variance (ANOVA) with delta speech MI in aSTG as dependent variable. As expected, the analysis yielded a significant main effect of alpha power, *F*(3,66) = 4.74, *p* < .01, *η*^2^ = .02, and a significant main effect of beta power, *F*(3,66)=8.25, *p*<.001, *η*^2^=.07. Furthermore, there was a significant alpha power×beta power interaction, *F*(9,66)=2.14, *p*=.03, *η*^2^=.02. The interaction suggests that frontal beta and central alpha power co-modulate auditory entrainment in aSTG. Furthermore, within-subject correlations between the power time series of frontal beta and central alpha were significant (mean *r*_Pearson_=.027, Student's *t*=-20.67, *p*<.001), and a cross-correlation showed that the correlation peaked with central alpha power following frontal beta power at a lag of 67 ms (95% CI [10,85], *p*=.02).

## 4 Discussion

The entrainment (alignment in time) of auditory cortex activity to speech is frequently used as a marker for how well the auditory brain encodes speech. Auditory entrainment has been linked with comprehension rates (e.g., Ahissar et al., 2001; Ding et al., 2014; Peelle and Davis, 2012) and is considered as a mechanistic component of sound encoding in the brain serving the segmentation and parsing of prosody, syllables or phonemes (e.g., Hyafil et al., 2015). In particular, entrainment in the theta band is often assumed to reflect the processing of the syllabic-scale information, as the syllabic rate of natural speech falls into the theta range (e.g., 6.8 Hz on average for the current speech stimulus) (Ding and Simon, 2014, Ghitza, 2012, Ghitza, 2013, Giraud and Poeppel, 2012, Hyafil et al., 2015, Peelle and Davis, 2012). In contrast, entrainment in the delta band has been proposed to reflect the processing of supra-segmental prosodic features such as acoustic stress, which are critical for the contextual processing and the parsing of speech (Ghitza, 2013, Ghitza et al., 2012, Giraud and Poeppel, 2012, Goswami and Leong, 2013, Greenberg et al., 2003). In line with this, a recent study demonstrated a direct influence of acoustic speech rhythm on auditory delta-entrainment (Kayser et al., 2015). It is noteworthy that, although entrainment is often associated with amplitude (i.e., intensity) fluctuations in the speech signal (e.g., Ahissar et al., 2001; Gross et al., 2013; Peelle and Davis, 2012), there is vast evidence that frequency (e.g., pitch) fluctuations are also tracked by the auditory system (Henry and Obleser, 2012, Henry and Obleser, 2013, Obleser et al., 2008, Obleser et al., 2012, Scott and McGettigan, 2012, Xu et al., 2005, Zeng et al., 2005). Particularly, spectral regularities in the delta band have been found to entrain neural oscillations (Henry and Obleser, 2012, Obleser et al., 2012) and could play a major role in the tracking of non-intensity based prosodic information that can contribute to speech comprehension.

Although neural entrainment has been extensively used to investigate the cortical processing of speech or other acoustic information, it is still under debate whether entrainment reflects a genuine oscillatory process (e.g., Zoefel and VanRullen, 2015) or a series of evoked potentials (Szymanski et al., 2011). Supporting the former view are, for example, findings that neural phase alignment in the delta-band can be found in the absence of acoustic fluctuations in the stimulus envelope, which is thought to reflect higher-level segmentations of speech (Ding et al., 2016; Meyer et al., 2016) or that entrainment can occur as the result of cross-modal attention (Lakatos et al., 2008, Lakatos et al., 2009).While research needs to resolve the exact neural mechanisms giving rise to rhythmic entrainment (see Kayser et al., 2015; Keitel, Quigley, & Ruhnau, 2014), it certainly serves as a powerful tool to index speech encoding (Giraud and Poeppel, 2012).

### 4.1 Network interactions indexed by oscillatory power

The auditory cortex functions within a large network of other temporal and fronto-parietal regions to provide the neural representations of speech. Many studies on speech networks have quantified the activity in fronto-parietal regions using the power of oscillatory activity, and have used this to attribute specific functions to these regions in speech encoding (Arnal et al., 2011, Bastiaansen and Hagoort, 2006, Bastiaansen et al., 2005, Obleser and Weisz, 2012, Obleser et al., 2012). Importantly, this often involves the implicit hypothesis that the processes indexed by fronto-parietal oscillatory power directly interact with the auditory cortical speech encoding implemented by rhythmic entrainment (Kayser et al., 2015). However, no study to date has systematically investigated whether and for which fronto-parietal brain regions the oscillatory activity directly interacts with auditory cortical speech entrainment.

We here provide a comprehensive analysis of such interactions and probe their temporal specificity to dissociate potential feed-forward from feed-back interactions. This revealed three auditory regions in which delta speech MI was systematically related to the oscillatory power in distinct clusters of fronto-parietal regions. Importantly, each of these network interactions was characterised by a distinct time scale of the relevant fronto-parietal state. Our results reveal a spatio-temporal organization whereby auditory delta-entrainment is influenced by frontal beta-activity, interacts bidirectionally with central alpha activity, and influences slow (theta) activity in parietal regions. By performing control analyses and excluding spatially proximal ROIs, we ruled out that these network interactions arise trivially from signal blurring in source space, general coupling mechanisms not related to the acoustic input, or a significant entrainment of fronto-parietal power to the speech signal itself. As we compare the auditory cortical entrainment to natural speech with a control condition of unintelligible (reversed) speech, our results do not allow conclusions about the immediate link between speech entrainment and speech comprehension, which have been investigated in many previous studies (e.g., Ding et al., 2014; Ding and Simon, 2013; Peelle et al., 2013). Rather, we here investigate the function of speech entrainment within a network context, and demonstrate how entrainment interacts with fronto-parietal networks. While future work is required to directly demonstrate the behavioural relevance of these interactions, we can speculate about hypothetical functions and their contribution to perception based on the specific oscillatory fingerprints associated with each of these.

As noted before, we used the power in different frequency bands as a marker of fronto-parietal activity. We thereby followed many previous studies that have used oscillatory power as index of possibly speech-relevant neural processes in various brain regions (e.g., Arnal and Giraud, 2012; Bastiaansen and Hagoort, 2006; Obleser and Weisz, 2012). Similarly, studies linking oscillatory activity to perception have shown that the power of pre-stimulus activity correlates with perceptual accuracy or reaction times (Arnal et al., 2015, Hanslmayr et al., 2007, Lundqvist et al., 2013, Ng et al., 2012, van Dijk et al., 2008). However, we acknowledge that many studies have also noted that the phase of oscillatory processes can correlate with perceptual outcomes or the quality of sound encoding (Henry et al., 2014, Henry and Obleser, 2012, Kayser et al., 2016, Ng et al., 2012). While it seems reasonable to repeat the present analysis using phase as a marker of the state of fronto-parietal activity, this is made difficult for several reasons. First, phase is technically well defined only when oscillatory power is sufficiently strong (Muthukumaraswamy and Singh, 2011). Second, the sign of source-localized activity is difficult to compare across regions and participants, given inherent ambiguities in the source localization process. This makes it difficult to assign an absolute interpretation to a specific phase angle in neuroimaging data, which is sometimes resolved with a within-subject standardization of phase angles for group-level analysis (Ng et al., 2013). In contrast, the linear relation between auditory entrainment and the power of oscillatory source activity described here can be easily compared and interpreted across regions and participants.

To date, only one study has provided indirect evidence for network-level influences on auditory cortical entrainment. Park et al. (2015) quantified a general measure of the functional connectivity between auditory cortex and the frontal lobe and found that the strength of top-down feedback from left precentral regions to auditory cortex correlates with the strength of entrainment across participants (Park et al., 2015). However, this study could neither implicate specific oscillatory processes in this top-down influence, nor did this study demonstrate that frontal activity directly affects the alignment of auditory activity to speech over time and within an individual participant.

### 4.2 Orbitofrontal beta and top-down predictions

We found evidence for a top-down modulation of delta-entrainment in the left aSTG by beta-power in predominantly left-lateralised medial orbitofrontal areas. Anatomically, orbitofrontal regions are connected with anterior superior temporal areas via a ventral stream (Gierhan, 2013). Medial frontal areas have been associated with story comprehension (Elliott et al., 2000, Maguire et al., 1999), phonological processing (Vigneau et al., 2006) and the generation of predictions (Bar, 2007, Dikker and Pylkkanen, 2013). The orbital gyrus has also been found to be involved in phrasal processing (Grodzinsky and Friederici, 2006). Beta oscillations have been suggested as a mechanism for the top-down propagation of predictions from higher to lower areas across the cortical hierarchy (Arnal and Giraud, 2012, Fontolan et al., 2014, Lewis and Bastiaansen, 2015) as well as the maintenance/change of the current mode of processing within the predictive coding framework (Bressler and Richter, 2015, Engel and Fries, 2010, Lewis et al., 2015). The involved areas, the frequency signature, and the direction of the interaction observed here are therefore consistent with a top-down scaling of auditory entrainment by predictions from frontal areas, whereby predictions facilitate the acoustic-phonological analysis within the aSTG (DeWitt and Rauschecker, 2012, Friederici, 2012). More specifically, increased beta power could indicate that the recent sensory input allowed the formation of clear predictions, while reduced beta power could indicate the lack of predictions about the expected acoustic input (Arnal and Giraud, 2012). The formation of strong predictions in prefrontal regions could then subsequently facilitate the dynamic representation of upcoming acoustic input in lower auditory regions, which is reflected by an enhancement of speech entrainment.

### 4.3 Auditory speech entrainment and central alpha power

Delta-entrainment in both left Heschl's Gyrus and aSTG was found to depend on alpha-power in bilateral central regions (pre- and postcentral gyri and SMA), extending to inferior parietal and median cingulate gyri. Motor areas are connected to auditory areas via the dorsal stream, and reflect articulatory mapping of heard speech (e.g., Friederici and Gierhan, 2013), and phoneme-specific activation (Pulvermüller et al., 2006). Furthermore, the SMA has been associated with temporal sensory prediction in auditory rhythm perception (Bengtsson et al., 2009, Grahn and Brett, 2007) and phoneme detection (Simon et al., 2002), while the cingulate gyrus has been associated with semantic processing (Zatorre et al., 1996) and phoneme detection (Simon et al., 2002). The alpha rhythm has a ubiquitous role in the brain as a gating mechanism ('functional inhibition', see Jensen and Mazaheri, 2010; Strauss et al., 2014) and has been found to indicate sensitivity to acoustic features in the context of speech intelligibility (Obleser and Weisz, 2012). Alpha is also one of the dominant rhythms in motor areas (e.g., Pineda, 2005). In line with this, we found that suppressed alpha-power in motor areas leads to enhanced delta-entrainment in early auditory areas. This might represent the recruitment of motor areas for a temporally precise phonological analysis in the Heschl's Gyrus and aSTG (DeWitt and Rauschecker, 2012, Friederici, 2012). While it seems unlikely that alpha power in central areas has a direct causal impact on auditory cortex, alpha may reflect the engagement of long-range connections that modulate the efficiency of information transmission along auditory pathways (Strauss et al., 2014) or which reflect the impact of task-engagement and cognitive effort on auditory cortex activity (Strauss et al., 2014, Wöstmann et al., 2015). Our data are ambivalent as to the direction of this interaction and previous work has revealed conflicting views on an automatic recruitment of motor cortex for auditory processing or the specifics of motor influences during high task demands (Alho et al., 2014, Morillon et al., 2015, Morillon et al., 2014). However, two recent studies have reported directed relations between left frontal activity and auditory cortical entrainment (Kayser et al., 2015, Park et al., 2015), cautiously supporting a top-down modulation.

### 4.4 Parietal theta power is modulated by speech entrainment in pSTG

The degree of delta-entrainment in the right posterior superior temporal gyrus negatively influenced theta-power in predominantly right-lateralised parietal areas, such as cuneus, precuneus, and superior/inferior parietal areas. The inferior parietal cortex is connected to the pSTG via dorsal streams (Gierhan, 2013). The right hemisphere, including parietal areas, has been associated with prosodic and intonational processing (Behrens, 1988, Behrens, 1989, Shapiro and Danly, 1985). Consistently, the right pSTG has been shown to be particularly involved in the processing of isolated prosody (Meyer et al., 2002). This right-hemisphere posterior network-level interaction might therefore represent the processing of prosodic information. Furthermore, theta-activity in the parietal cortex is usually associated with working-memory processes, where theta-power increases with working-memory load (Moran et al., 2010, Raghavachari et al., 2001, Sauseng et al., 2010). It has also been proposed that theta power changes during language processing reflect lexical retrieval processes and semantic working memory (Bastiaansen and Hagoort, 2006, Bastiaansen et al., 2005). We found that weaker auditory entrainment leads to stronger theta power in parietal regions. One plausible interpretation of the present result is that weak delta-parsing is subsequently compensated by parietal working memory processes that support linguistic inference. In other words, weak entrainment, and the likely resulting poor comprehension (e.g., Doelling et al., 2014), might necessitate stronger working memory involvement to make sense of the heard speech.

### 4.5 The network interactions of auditory entrainment

We did not observe any significant interactions of auditory theta band entrainment with fronto-parietal activity. During listening to the naturally-told comedy story used as stimulus here, auditory activity entrains to the speech envelope both in the delta and theta bands, but entrainment is stronger in the delta band (see also, Gross et al., 2013). One possible explanation for this delta dominance is that our acoustic stimulus is rich in prosodic features, in contrast to more neutral speech such as sequences of digits (Doelling et al., 2014, Ghitza, 2012) or individual sentences (Peelle et al., 2013). Importantly, entrainment in these frequency bands can directly dissociate in the same experimental paradigm (Ding et al., 2014, Kayser et al., 2015). Across multiple studies, it has been observed that changes in theta band entrainment correlate with changes in acoustic features such as the signal-to-noise ratio or voice vocoding (Ding et al., 2014, Ding and Simon, 2013, Peelle et al., 2013). It has therefore been tied to acoustic speech properties (Ding and Simon, 2013). On the other hand, delta-entrainment correlates more strongly with the perceived quality of speech or intelligibility measures (Ding et al., 2014, Ding and Simon, 2013) and has also been implied in attentional selection (Ding and Simon, 2013, Schroeder and Lakatos, 2009) and the formation of temporal predictions (Arnal et al., 2015). In addition, two recent studies show that auditory delta oscillations modulate the interpretation of linguistic input (Meyer et al., 2016), and can track hierarchical linguistic structures even without the presence of acoustic cues (Ding et al., 2016). Thus, there seems to be converging evidence that delta oscillations are involved in higher order cognitive functions during speech processing such as attention, semantic comprehension and lexical interpretation. These may be reasons for why long-range network interactions of auditory entrainment are more prominent for delta than for theta entrainment.

**Fig. 5:**
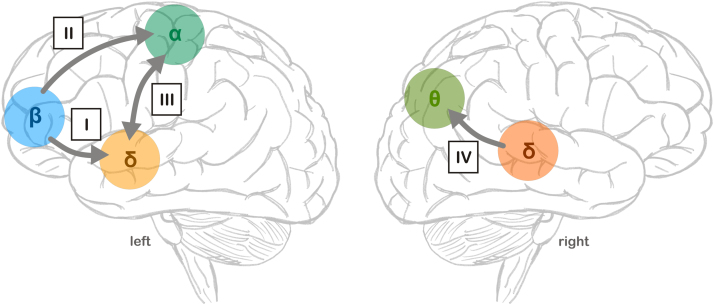
Summary and time course of network-level effects of delta-entrainment in auditory areas. *Left hemisphere*: I) Prefrontal predictions facilitate delta-speech tracking in aSTG. II) Frontal predictions are conveyed to motor areas. III) Motor areas interact with delta-entrainment in aSTG. *Right hemisphere*: IV) Delta-speech tracking in pSTG engages parietal areas. aSTG: anterior superior temporal gyrus, pSTG posterior superior temporal gyrus.

However, our results suggest a cascade of network mechanisms that control the parsing of continuous, natural speech by delta entrainment (see Fig. 5). In the left hemisphere, orbitofrontal areas influence the alignment of rhythmic auditory cortical activity to speech, possibly by providing predictive information. These predictions are also conveyed to the motor system, as shown by a significant cross-correlation between frontal beta power and central alpha. The motor system in turn interacts with delta-entrainment, possibly by exploiting the frontal predictions and utilising its ability to process rhythms in a temporally precise manner. Finally, in the right hemisphere, parietal theta processes are particularly engaged in moments when auditory delta-entrainment is weak. As we did not employ any direct experimental manipulation or the collection of behavioural measures, these interpretations necessarily remain speculative. However, our results directly place the entrainment of rhythmic auditory cortical activity in the context of fronto-parietal speech relevant networks. They provide a basis to further explore the functional and possibly mechanistic relations between auditory cortical entrainment, semantic and lexical processes at higher stages of the auditory pathways, and perception.

## Funding

This work was supported by the UK Biotechnology and Biological Sciences Research Council (BBSRC, BB/L027534/1); a European Research Council grant (ERC-2014-CoG; Grant No 646657) to CK; and a Wellcome Trust grant (Joint Senior Investigator Award, No 098433) to JG.
